# Supplementary material for: Progress on the Link between Nutrient Availability and Toxin Production by Ostreopsis cf. ovata: Field and Laboratory Experiments
Source: Toxins (Basel). 2023 Mar 2;15(3):188. doi: 10.3390/toxins15030188 (PMC10057244; doi:10.3390/toxins15030188)
Supplement: Supplementary file 1 [file toxins-15-00188-s001.zip › toxins-2181620-supplementary.pdf]

toxins

Supplementary Materials

Progress on the Link between Nutrient Availability and Toxin Production by *Ostreopsis* cf. *ovata*: Field and Laboratory Experiments

Noemí Inmaculada Medina-Pérez [1,2], Elena Cerdán-García [3], Francesc Rubió [2], Laia Viure [2], Marta Estrada [2], Encarnación Moyano [1,4] and Elisa Berdalet [2,*]

[1] Department of Chemical Engineering and Analytical Chemistry, University of Barcelona. Av. Diagonal 645, E-08028 Barcelona, Spain
[2] Department of Marine Biology and Oceanography, Institute of Marine Sciences (ICM-CSIC), Pg. Marítim de la Barceloneta, 37-49, E-08003 Barcelona, Spain
[3] Aix Marseille Université, Université de Toulon, CNRS, IRD, MIO UM 110, 13288, Marseille, France
[4] Water Research Institute (IdRA), University of Barcelona, Montalegre 6, E-08001 Barcelona, Spain

- **Table S1:** Toxin profiles (cellular content of the different PLTX analogues expressed as % of the total toxin cell content) detected in the samples **(a)** obtained throughout the *Ostreopsis* cf. *ovata* 2018 bloom and **(b)** in the different growth phases of the experimental cultures (E = Exponential; I = Intermediate; S = Stationary). The corresponding pie charts are shown in Figure 3.

- **Table S2:** Culture growth phase corresponding to the toxin and biovolume sampling days (indicated by numbers) for the different experimental conditions. The Exponential, Intermediate, and Stationary phases were identified based on the growth curves plotted in Figure 4. Details of the experimental design are shown in Figure S2.

- **Table S3:** Average, standard deviation, and median (in all cases n=100) of the DV measured in the different phases of the growth curves (Exponential, Intermediate, and Stationary) of each experimental treatment. The Exponential, Intermediate, and Stationary phases were identified based on the growth curves plotted in Figure 4. Details of the experimental design are shown in Figure S2.

- **Figure S1:** Pearson correlation coefficients between all the variables measured in the field during the 2018 *O.* cf. *ovata* bloom. N: nitrogen, P: phosphorus, TN: total nitrogen, TP: total phosphorus. TOC: total organic carbon. The critical value for $p<0.05$ (two-tailed test) and 11 degrees of freedom (n-2) is 0.55.

- **Figure S2:** Scheme of the design of experimental series 1 and 2. The replicate flasks are labeled from I to V. t_i indicates the sampling day for each experimental condition as described in Section 5.2.

toxins

Table S1: Toxin profiles (cellular content of the different PLTX analogues expressed as % of the total toxin cell content) detected in the samples **(a)** obtained throughout the *Ostreopsis* cf. *ovata* 2018 bloom and **(b)** in the different growth phases of the experimental cultures (E = Exponential; I = Intermediate; S = Stationary). The corresponding pie charts are shown in Figure 3.

a) Toxin profiles in the *Ostreopsis* cf. *ovata* bloom samples (two replicate filters per sampling day)

Date	Julian day	OVTX-a	OVTX-b	OVTX-c	OVTX-d	OVTX-e	OVTX-f	OVTX-g	IsoPLTX
2018/06/21	172								
2018/07/05	186								
2018/07/10	191								
2018/07/20	201	66.5±0.4	21.8±0.4	2.8±0.1	6.2±0.1	2.7±0.6			
2018/07/24	205	67.4±0.1	20.1±0.7	2.2±0.1	5.0±0.1	4.3±0.1	0.9±0.2	0.7±0.1	0.4±0.1
2018/07/27	208	66.2±0.9	20.0±0.3	2.1±0.1	4.9±0.1	5.6±0.2		0.9±0.2	0.8±0.1
2018/08/01	213	67.6±0.3	22.2±0.4	2.0±0.1	4.1±0.8	3.8±0.4			0.7±0.1
2018/08/08	220	69.2±2.2	30.8±2.2						
2018/08/21	233	68.1±1.1	24.2±0.5	2.6±0.1	3.8±0.5	2.6±0.1			
2018/08/28	240	71.9±3.3	28.1±3.3						
2018/09/13	256	64.3±0.2	25.6±0.2	3.9±0.5	3.9±0.5	1.3±0.1	2.0±0.1		
2018/10/03	276	69.8±0.1	30.2±0.1						
2018/06/21	311								

b) Toxin profiles in the experiments, by treatment and growth phase; the number of filters (n) varied as detailed in Table S2.

Treatment	Phase	OVTX-a	OVTX-b	OVTX-c	OVTX-d	OVTX-e	OVTX-f	OVTX-g	IsoPLTX
f/2	E (n=4)	55.2±3.2	26.3±1.4	6.8±0.2	8.9±1.8	2.9±1.8			
	I (n=4)	60.4±1.2	23.7±0.6	4.1±0.3	6.5±0.4	4.6±0.1			0.58±0.05
	S (n=13)	40.2±5.1	26.8±4.9	7.6±1.9	16.0±4.1	6.7±1.2	0.5±0.1	1.0±0.4	1.2±0.3
Control	E (n=4)	56.9±2.5	24.8±0.8	8.3±1.3	10.0±1.5				
	I (n=4)	59.9±1.2	24.6±0.9	4.1±0.1	6.5±0.3	4.4±0.1			0.51±0.05
	S (n=13)	53.4±5.3	27.2±2.8	4.4±0.5	9.1±1.6	4.9±0.6	0.4±0.1	0.6±0.1	0.7±0.1
P-deficiency	E (n=4)	55.9±1.2	27.3±0.5	5.1±0.4	8.1±0.7	3.7±0.7			
	S (n=17)	54.0±5.8	27.0±4.5	4.6±0.4	8.2±0.8	5.2±0.6	0.5±0.1	0.8±0.4	0.6±0.2
N-deficiency	E (n=4)	56.8±1.6	26.8±1.3	5.1±0.5	8.2±0.5	4.2±0.3			
	S (n=17)	58.4±4.2	23.4±5.0	4.4±0.5	8.2±0.9	5.2±0.6			0.5±0.3

Nitrate 50 µM	E (n=3)	57.3±2.1	26.5±1.5	4.3±0.2	7.4±0.4	4.5±0.2			
	I (n=3)	48.3±1.0	33.5±0.4	4.9±0.2	8.5±0.5	4.8±0.3			
	S (n=9)	49.6±2.6	23.9±0.9	7.2±0.9	10.9±1.3	7.6±0.7	0.3±0.1	0.3±0.1	0.7±0.3
Ammonium 50 µM	E (n=3)	49.5±13.5	22.2±4.6	3.9±0.6	6.9±0.1	3.7±0.7			
	I (n=3)	48.5±1.6	31.4±2.5	5.0±0.6	9.9±2.7	4.7±0.6			0.5±0.4
	S (n=9)	50.6±3.2	26.4±2.0	5.9±0.5	10.4±1.9	5.8±0.6	0.3±0.2	0.3±0.2	0.6±0.1
Urea 50 µM	E (n=3)	53.9±2.0	28.6±2.0	5.5±0.1	10.3±1.1	5.5±0.2			
	I (n=3)	55.7±0.8	27.2±0.7	4.4±0.1	8.3±0.3	4.4±0.5			
	S (n=9)	49.0±4.3	25.1±2.0	6.5±1.2	12.1±3.1	6.3±1.0	0.3±0.1	0.3±0.1	0.8±0.1
Fertilizer 50 µM	E (n=3)	56.1±2.7	26.6±1.4	4.7±0.4	7.6±0.5	5.0±0.5			
	I (n=3)	54.5±4.1	29.4±4.4	4.4±0.1	7.3±0.2	4.4±0.2			
	S (n=9)	48.2±1.7	30.6±1.3	4.7±0.2	10.2±2.0	5.2±0.3	0.6±0.3	0.6±0.3	0.2±0.3
Nitrate 0.5 µM	E (n=2)	56.7±0.2	31.6±0.1		11.8±0.1				
	I (n=2)	56.5±0.5	31.8±0.6		11.7±0.1				
	S (n=2)	55.3±3.1	29.9±1.3	4.9±0.1	9.6±0.7	5.4±0.1			
Ammonium 0.5 µM	E (n=8)	47.8±1.0	28.7±0.7	7.3±0.2	12.7±1.0	9.5±1.5			
Urea 0.5 µM	E (n=6)	57.4±3.1	32.4±1.9		10.9±0.7	6.2±0.2			
Fertilizer 0.5 µM	E (n=2)	50.6±0.9	29.4±0.3		11.5±0.6	8.6±0.6			
	I (n=2)	51.3±1.3	28.5±0.9	6.2±0.2	10.0±0.3	7.1±0.6			
	S (n=2)	46.7±4.0	31.0±2.9	5.9±0.3	10.2±0.6	6.1±0.2			

Table S2: Culture growth phase corresponding to the toxin and biovolume sampling days (indicated by numbers) for the different experimental conditions. The Exponential, Intermediate, and Stationary phases were identified based on the growth curves plotted in Figure 4. Details of the experimental design are shown in Figure S2.

	Growth culture phase		
**Treatments**	**Exponential**	**Intermediate**	**Stationary**
**f/2**	2	7	18, 30
**Control**	2	7	18, 30
**P-deficiency**	2	-	7, 18, 30
**N-deficiency**	2	-	7, 18, 30
**Nitrate 50 µM**	2	4	7, 10, 15
**Ammonium 50 µM**	2	4	7, 10, 15
**Urea 50 µM**	2	4	7, 10, 15
**Fertilizer 50 µM**	2	4	7, 10, 15
**Nitrate 0.5 µM**	2	4	7
**Ammonium 0.5 µM**	2, 4, 7	-	-
**Urea 0.5 µM**	2, 4, 7	-	-
**Fertilizer 0.5 µM**	2	4	7

Table S3: Average, standard deviation, and median (in all cases n=100) of the DV measured in the different phases of the growth curves (Exponential, Intermediate, and Stationary) of each experimental treatment. The Exponential, Intermediate, and Stationary phases were identified based on the growth curves plotted in Figure 4. Details of the experimental design are shown in Figure S2.

	Exponential DV (µm)		Intermediate DV (µm)		Stationary DV (µm)	
	**avg ± std**	**median**	**avg ± std**	**median**	**avg ± std**	**median**
**f/2**	47±5	47	42±6	42	40±7	39
**Control**	55±5	55	56±6	57	56±7	57
**P-deficiency**	56±6	56	-	-	54±9	56
**N-deficiency**	53±6	53	-	-	53±7	53
**Nitrate 50 µM**	42±7	43	40±8	39	41±8	39
**Ammonium 50 µM**	42±8	43	40±9	38	41±10	41
**Urea 50 µM**	32±5	31	31±6	30	32±6	31
**Fertilizer 50 µM**	40±7	42	37±7	37	37±7	35
**Nitrate 0.5 µM**	36±7	34	35±6	34	36±6	35
**Ammonium 0.5 µM**	33±7	32	-	-	-	
**Urea 0.5 µM**	35±6	34	-	-	-	
**Fertilizer 0.5 µM**	35±8	33	36±7	35	34±5	34

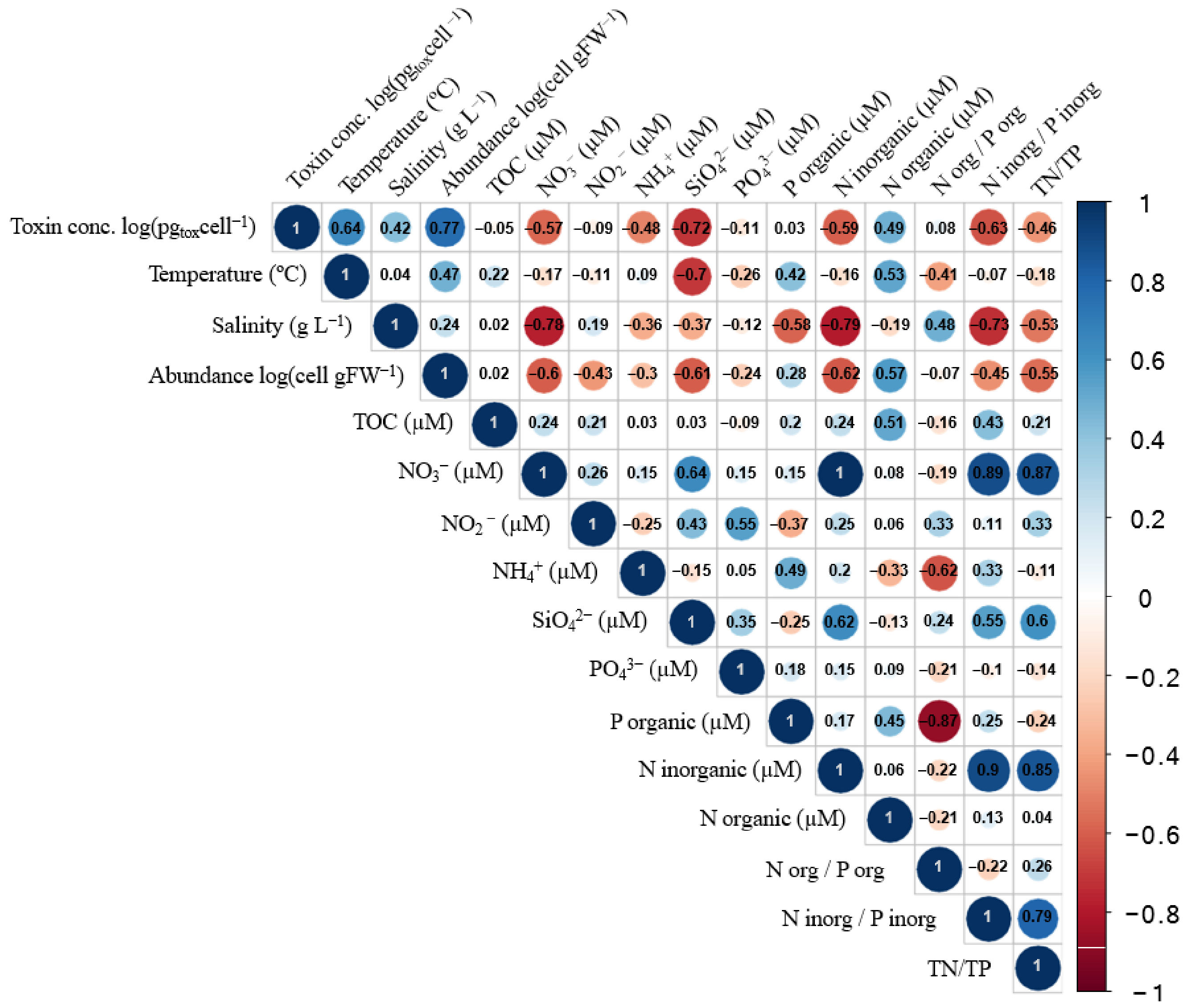

Figure S1: Pearson correlation coefficients between all the variables measured in the field during the 2018 *O.* cf. *ovata* bloom. N: nitrogen, P: phosphorus, TN: total nitrogen, TP: total phosphorus. TOC: total organic carbon. The critical value for $p<0.05$ (two-tailed test) and 11 degrees of freedom (n-2) is 0.55.

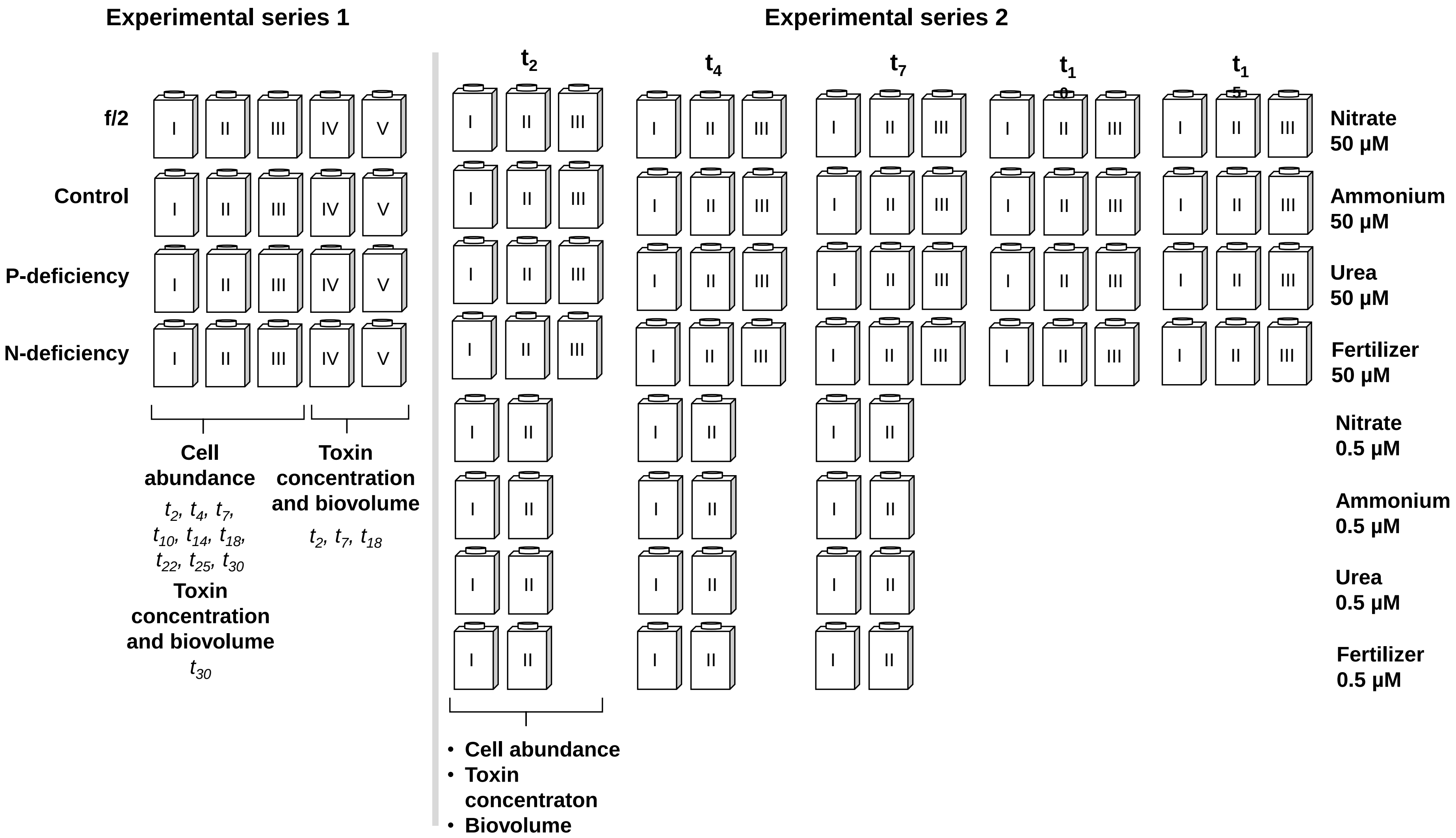

Figure S2: Scheme of the design of experimental series 1 and 2. The replicate flasks are labeled from I to V. t_i indicates the sampling day for each experimental condition as described in Section 5.2.
